# Locomotor Muscle Fatigue Does Not Alter Oxygen Uptake Kinetics during High-Intensity Exercise

**DOI:** 10.3389/fphys.2016.00463

**Published:** 2016-10-13

**Authors:** James G. Hopker, Giuseppe Caporaso, Andrea Azzalin, Roger Carpenter, Samuele M. Marcora

**Affiliations:** ^1^Endurance Research Group, School of Sport and Exercise Sciences, University of KentChatham, UK; ^2^Applied Sport Science Research Group, School of Health, Sport and Bioscience, University of East LondonLondon, UK; ^3^Leicester City Football ClubLeicester, UK

**Keywords:** locomotor muscle fatigue, slow component, efficiency, power output, aerobic exercise, cycling

## Abstract

The $\dot{\text{V}}{\text{O}}_{2}$ slow component ($\dot{\text{V}}{\text{O}}_{2^{\text{sc}}}$) that develops during high-intensity aerobic exercise is thought to be strongly associated with locomotor muscle fatigue. We sought to experimentally test this hypothesis by pre-fatiguing the locomotor muscles used during subsequent high-intensity cycling exercise. Over two separate visits, eight healthy male participants were asked to either perform a non-metabolically stressful 100 intermittent drop-jumps protocol (pre-fatigue condition) or rest for 33 min (control condition) according to a random and counterbalanced order. Locomotor muscle fatigue was quantified with 6-s maximal sprints at a fixed pedaling cadence of 90 rev·min^−1^. Oxygen kinetics and other responses (heart rate, capillary blood lactate concentration and rating of perceived exertion, RPE) were measured during two subsequent bouts of 6 min cycling exercise at 50% of the delta between the lactate threshold and $\dot{\text{V}}{\text{O}}_{2^{\text{max}}}$ determined during a preliminary incremental exercise test. All tests were performed on the same cycle ergometer. Despite significant locomotor muscle fatigue (*P* = 0.03), the $\dot{\text{V}}{\text{O}}_{2^{\text{sc}}}$ was not significantly different between the pre-fatigue (464 ± 301 mL·min^−1^) and the control (556 ± 223 mL·min^−1^) condition (*P* = 0.50). Blood lactate response was not significantly different between conditions (*P* = 0.48) but RPE was significantly higher following the pre-fatiguing exercise protocol compared with the control condition (*P* < 0.01) suggesting higher muscle recruitment. These results demonstrate experimentally that locomotor muscle fatigue does not significantly alter the $\dot{\text{V}}{\text{O}}_{2}$ kinetic response to high intensity aerobic exercise, and challenge the hypothesis that the $\dot{\text{V}}{\text{O}}_{2^{\text{sc}}}$ is strongly associated with locomotor muscle fatigue.

## Introduction

The oxygen uptake ($\dot{\text{V}}{\text{O}}_{2}$) kinetics during aerobic exercise is characterized by a tri-phasic response profile (Whipp and Ward, 1990). Following a short time delay (phase I) (~20 s mainly reflecting increased pulmonary perfusion and changes in lung gas stores), pulmonary $\dot{\text{V}}{\text{O}}_{2}$ rises in a mono-exponential fashion (phase II) leading to the attainment of steady-state oxygen uptake within 2–3 min (phase III). During exercise above the lactate threshold (LT), but below $\dot{\text{V}}{\text{O}}_{2^{\text{max}}}$, the exponential kinetics are complicated by an additional component resulting in a slow rise $\dot{\text{V}}{\text{O}}_{2}$ above that expected from the sub-LT linear relationship between $\dot{\text{V}}{\text{O}}_{2}$ and power output. This phase, termed the $\dot{\text{V}}{\text{O}}_{2}$ “slow component” (Whipp, 1994), is a manifestation of reduced muscle efficiency during exercise above LT. During exercise above the “critical power,” the $\dot{\text{V}}{\text{O}}_{2}$ slow component ($\dot{\text{V}}{\text{O}}_{2^{\text{sc}}}$) will cause $\dot{\text{V}}{\text{O}}_{2}$ to rise continuously until $\dot{\text{V}}{\text{O}}_{2^{\text{max}}}$ and eventually volitional exhaustion is reached (Murgatroyd et al., 2011).

The $\dot{\text{V}}{\text{O}}_{2^{\text{sc}}}$ that develops during high-intensity aerobic exercise is thought to be strongly associated with locomotor muscle fatigue (defined as an exercise-induced reduction in maximal voluntary force or power produced with the locomotor muscles) because of shared mechanisms like decreased “metabolic stability,” muscle metabolite accumulation, decreased free energy of ATP breakdown, limited O_2_ or substrate availability, increased glycolysis, pH disturbance, increased muscle temperature, ROS production, and altered motor unit recruitment patterns (Grassi et al., 2015). However, empirical evidence of such association is sparse. In a seminal study, Cannon et al. (2011) quantified locomotor muscle fatigue induced by cycling exercise at various intensities (80% LT, 20 and 60% Δ) using 5-s isokinetic sprints to measure maximal voluntary cycling power (MVCP) at three different pedaling cadences (60, 90, and 120 rev·min^−1^). They found that, across exercise intensities, the overall reduction in MVCP (i.e., the magnitude of locomotor muscle fatigue) was significantly correlated with the magnitude of the $\dot{\text{V}}{\text{O}}_{2^{\text{sc}}}$. This finding suggests that locomotor muscle fatigue might be a pre-requisite for the development of the $\dot{\text{V}}{\text{O}}_{2^{\text{sc}}}$ observed during high-intensity aerobic exercise. Indeed, previous research has suggested the $\dot{\text{V}}{\text{O}}_{2^{\text{sc}}}$ might be caused by the progressive recruitment of less-efficient Type II muscle fibers in order to compensate for locomotor muscle fatigue and maintain power output during high-intensity cycling exercise (Barstow et al., 1996; Krustrup et al., 2004b; Endo et al., 2007). However, Cannon et al. (2011) observed that locomotor muscle fatigue occurs early and does not progress further between the 3rd and 8th min of cycling exercise above LT despite the substantial $\dot{\text{V}}{\text{O}}_{2^{\text{sc}}}$ developing during this period of time. Therefore, additional recruitment of less-efficient type II muscle fibers to compensate for locomotor muscle fatigue might not necessarily be required for the development of the $\dot{\text{V}}{\text{O}}_{2^{\text{sc}}}$ during high-intensity aerobic exercise. This temporal dissociation between locomotor muscle fatigue and the $\dot{\text{V}}{\text{O}}_{2^{\text{sc}}}$ during high-intensity aerobic exercise suggests that their correlation across exercise intensities may be a spurious one. Specifically, it has been suggested that an increased ATP and/or O_2_ cost of power production is responsible for the $\dot{\text{V}}{\text{O}}_{2^{\text{sc}}}$ observed during exercise above LT (Cannon et al., 2011). Therefore, the difference in metabolic stress between different exercise intensities, not locomotor muscle fatigue itself, may explain the correlation with the $\dot{\text{V}}{\text{O}}_{2^{\text{sc}}}$ observed by Cannon et al. (2011). Moreover, data from Vanhatalo et al. (2011) demonstrates that during a 3 min all-out cycling test, the development of the $\dot{\text{V}}{\text{O}}_{2^{\text{sc}}}$ was not associated with progressive muscle fiber recruitment. Instead, the authors suggest that $\dot{\text{V}}{\text{O}}_{2^{\text{sc}}}$ was the result of a higher oxygen cost per unit of external work done by the fatigued fibers, i.e., a reduction in muscle efficiency.

In the present study, we used an experimental design to test the hypothesis that a cause-and-effect relationship exists between locomotor muscle fatigue and $\dot{\text{V}}{\text{O}}_{2}$ kinetics during high-intensity aerobic exercise. In order to isolate the effects of reduced maximal voluntary power from the confounding effects of metabolic stress, we induced locomotor muscle fatigue using the 100 intermittent drop jumps protocol originally developed by Skurvydas et al. (2000). This 100 intermittent drop jumps protocol is known to cause prolonged locomotor muscle fatigue by disrupting the excitation:contraction coupling machinery primarily in Type II muscle fibers (Nielsen et al., 2005). Furthermore, the 20-s rest period after each drop jump allows for recovery, through oxidative phosphorylation, of the ATP and phosphocreatine expended during each jump (Nielsen et al., 2005). As a result, significant locomotor muscle fatigue can be induced without the metabolic stress associated with high-intensity aerobic exercise. In this way, we could experimentally isolate the effects of having to increase muscle recruitment to compensate for locomotor muscle fatigue (operationally defined as an exercise-induced reduction in MVCP) from the effects of other mechanisms like muscle metabolite accumulation, limited O_2_ or substrate availability, increased glycolysis and pH disturbance on $\dot{\text{V}}{\text{O}}_{2}$ kinetics during high-intensity cycling exercise.

## Materials and methods

### Participants

We recruited eight male participants (mean ± SD: age 24 ± 5 yr, body mass 76 ± 8 kg, height 1.81 ± 0.54 m, $\dot{\text{V}}{\text{O}}_{2^{\text{peak}}}$ 45.6 ± 6.5 mL·kg^−1^·min^−1^) for this study. All participants were screened for known heart or respiratory disease by completing a health and physical activity readiness questionnaire. The study received institutional ethics approval in accordance with the Declaration of Helsinki. All participants provided written informed consent before participating.

### Study protocol

Each participant completed three visits. During the first visit, participants first completed an incremental exercise test to determine LT and $\dot{\text{V}}{\text{O}}_{2^{\text{peak}}}$. A smooth ramped-incremental test protocol (Lode Excalibur Sport, Groningen, Netherlands) was used, which started at 100 W and increasing by an equivalent of 20 W per minute until the participant could no longer maintain a cadence of at least 60 rev. min^−1^ at the specified power output, despite strong verbal encouragement. Pulmonary gas exchange and ventilation was measured breath-by-breath. Participants wore a flexible facemask that fully covered mouth and nose and breathed through a mouthpiece and impeller turbine assembly. Before each test, the gas analyzer (MetaMax 3B, Cortex Biophysik, Leipzig, Germany) was calibrated for gas and volume measurements, according to the manufacturer's guidelines. The gas analyser was calibrated with gases of known concentrations over the span expected during exercise, and the turbine volume transducer was calibrated using a 3-L syringe over a range of different flow rates (Hans Rudolph, MO). $\dot{\text{V}}{\text{O}}_{2^{\text{peak}}}$ was recorded as the highest exercise test mean oxygen consumption over a 30-s period. The LT was estimated as the $\dot{\text{V}}{\text{O}}_{2}$ equivalent to: (1) a non-linear increase in VCO_2_ output, compared to $\dot{\text{V}}{\text{O}}_{2}$; (2) an increase in the ventilatory equivalent for oxygen (V_E_/ $\dot{\text{V}}{\text{O}}_{2}$), without an increase in V_E_/VCO_2_; (3) an increase in end-tidal partial pressure of O_2_ (P_ET_O_2_) without a fall in the end-tidal partial pressure of CO_2_ (P_ET_CO_2_) (Beaver et al., 1986).

After a short rest, participants were familiarized with the maximal voluntary cycling power test (MVCP) used during the subsequent two visits. Participants were required to sprint maximally for 6 s on an SRM ergometer (SRM, Julich, Germany) set in “isokinetic mode” with a fixed pedaling cadence of 90 rev·min^−1^. This was repeated a further two times for the purposes of familiarization.

**Figure 1:**
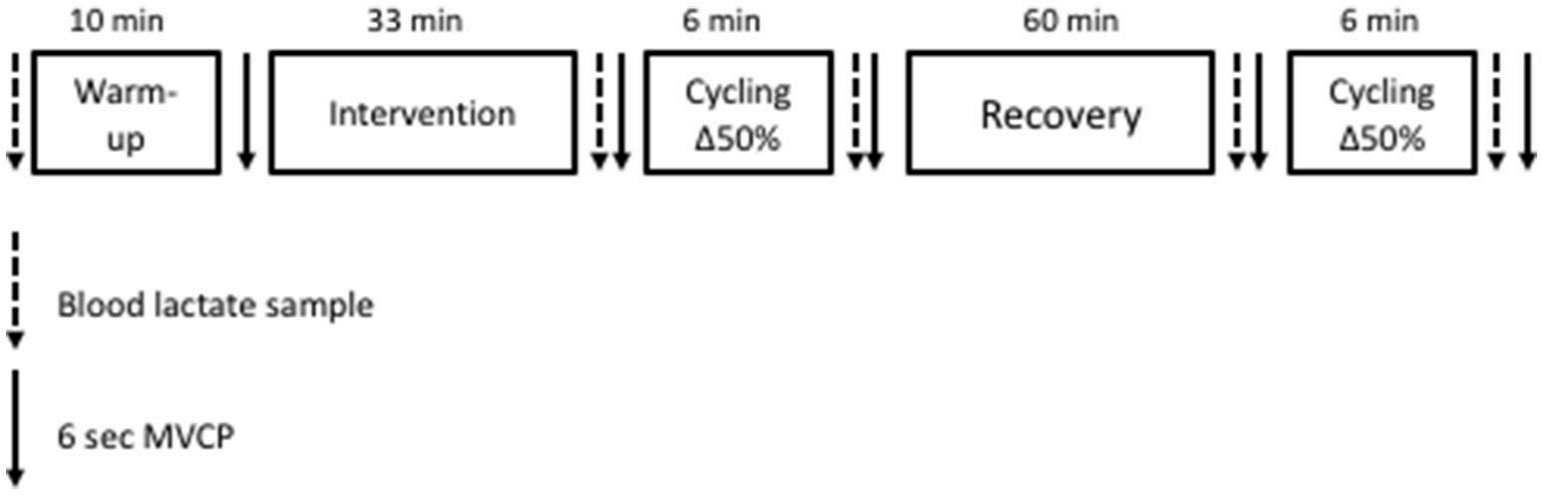
**Experimental visits protocol**.

Participants completed two further experimental visits (Figure 1). Each visit commenced with a baseline finger prick blood lactate sample (Biosen C-Line, EKF Diagnostics, London, UK) and 10-min warm up at 100 W at 90 rev·min^−1^ following which participants performed a MVCP test. MVCP at an isokinetic pedaling cadence of 90 rev·min^−1^ was taken as the highest power output recorded during the 6-s sprint. Power data was recorded at the crank of the ergometer once every revolution throughout the MVCP test. Prior to each test, the SRM ergometer was calibrated according to the manufacturer recommendations. Next, participants were asked to either perform the 100 intermittent drop-jumps protocol (pre-fatigue condition) or rest for 33 min (control condition) according to a random and counterbalanced order. In the pre-fatigue condition, participants dropped 100 times from a 40-cm high platform down to 90° knee angle before jumping upward as high as possible. Between each jump there was a 20-s rest period. Marcora et al. (2008) observed no increase in capillary blood lactate concentration after this fatiguing exercise protocol, which requires only moderate cardiovascular strain (58% of maximum heart rate) for 33 min. The control condition consisted of resting comfortably for 33 min. Two minutes after completing the allocated experimental manipulation, another finger prick blood lactate sample was taken before a second MVCP test was performed. Two minutes following the MVCP, participants then completed a bout of 6 min high-intensity cycling exercise to assess the $\dot{\text{V}}{\text{O}}_{2}$ kinetic response. This exercise protocol consisted of 2 min rest, sitting on the ergometer (Lode Excalibur Sport, Groningen, Netherlands), 3 min of “unloaded” cycling at 90 rev·min^−1^, and finally a transition to a power output calculated to require 50% delta (Δ50%). The power output at Δ50% was calculated as the difference between estimated LT and $\dot{\text{V}}{\text{O}}_{2^{\text{peak}}}$ using the formula: Δ50% = LT + 0.5 × ($\dot{\text{V}}{\text{O}}_{2^{\text{peak}}}$ – LT). Following the bout of high-intensity cycling exercise, another finger prick blood lactate sample was taken before a third MVCP test was performed. Participants then rested for 60 min before repeating the exercise protocol described above to allow replication of the $\dot{\text{V}}{\text{O}}_{2}$ kinetic response. Due to the long lasting nature of fatigue induced by the intermittent jumping protocol (Twist and Eston, 2005) it was not deemed necessary for participants to complete a further bout of fatiguing exercise prior to the 2nd period cycling at Δ50%. Before and after this second bout of high-intensity cycling exercise, a finger prick blood lactate sample was taken before participants performed a MVCP test.

Heart rate was measured throughout all exercise tests using a monitor (Polar Electro Oy, Kempele, Finland) with a 1 Hz sampling frequency, and then averaged over each minute of the 6 min test. Rating of perceived exertion (RPE) was taken each minute using the Borg RPE scale and memory anchoring procedures (Borg, 1998). In both studies, all visits were completed within 21 days of the first visit, and at least 7 days apart and at the same time of day. Participants were instructed to arrive at the laboratory well hydrated and having abstained from intense exercise, alcohol and caffeinated beverages within the 24 h preceding each visit.

### Oxygen uptake kinetics analysis

The breath-by-breath $\dot{\text{V}}{\text{O}}_{2}$ from each test were initially examined to exclude errant breaths caused by coughing, swallowing, sighing, etc., and those values lying more than four standard deviations from the local mean were removed. The breath-by-breath data were subsequently linearly interpolated to provide second-by-second values and, for each individual, the two repetitions of the Δ50% exercise were time-aligned to the start of exercise and ensemble averaged. The first 20 s of data after the onset of exercise were deleted to ensure the exclusion of the cardiopulmonary phase (Whipp and Rossiter, 2005) and a non-linear least-square algorithm was used to fit the data thereafter. In this equation $\dot{\text{V}}{\text{O}}_{2}$ baseline indicates the baseline value obtained during the 3 min of “unloaded” cycling at 90 rev·min^−1^, calculated as the mean $\dot{\text{V}}{\text{O}}_{2}$ measured over the final 60 s unloaded cycling. A bi-exponential model was used to characterize the response kinetics in its constituent fundamental and slow components. Responses from the two transitions in each condition were ensemble averaged to improve signal-to-noise ratio and fitted according to the following function:

$$\begin{array}{l} \dot{\text{V}}{\text{O}}_{2}\left(\text{t}\right)=\dot{\text{V}}{{\text{O}}_{2}}_{\text{b}}+{\text{A}}_{\text{p}}\left(1-\text{e}-\left(\text{t}-{\text{TD}}_{\text{p}}/\tau_{\text{p}}\right)\right)\\ +{\text{A}}_{\text{sc}}\left(1-\text{e}-\left(\text{t}-{\text{TD}}_{\text{sc}}/\tau_{\text{sc}}\right)\right) \end{array}$$

where $\dot{\text{V}}{\text{O}}_{2}$ (*t*) represents the absolute $\dot{\text{V}}{\text{O}}_{2}$ at a given time *t*; $\dot{\text{V}}{\text{O}}_{2\text{b}}$ represents the mean $\dot{\text{V}}{\text{O}}_{2}$ in the baseline period; A_p_, TD_p_, and τ_p_ represent the amplitude, time delay, and time constant, respectively, describing the fundamental or phase II increase in $\dot{\text{V}}{\text{O}}_{2}$ above baseline; and A_sc_, TD_sc_, and τ_sc_ represent the amplitude of, time delay before the onset of, and time constant describing the development of, the $\dot{\text{V}}{\text{O}}_{2}$ slow component, respectively. Data fitting were carried out by using a commercial available software (GraphPad Prism 4, GraphPad Software).

### Statistical analysis

The statistical software package SPSS was used for all statistical analysis (Version 14.0, SPSS, Chicago, Illinois). Prior to analysis all data was checked for normality of distribution using a Shapiro-Wilk test. The effects of condition (pre-fatigue vs. control) on parameters associated with the $\dot{\text{V}}{\text{O}}_{2}$ kinetic response was assessed using paired *t*-tests. Two-way repeated measures ANOVAs (condition × time) were used to analyse the MVCP, blood lactate concentration. Heart rate and RPE data were analyzed by two-way repeated measures MANOVA (condition × bout × time). For all repeated measures analysis, if a significant interaction was revealed, the main effect of condition was not considered, and tests of simple main effects of condition were conducted as follow-up using the Least significant difference *post*-*hoc* test. Statistical significance was accepted at *p* < 0.05. Results are reported as mean ± SD unless otherwise stated.

## Results

### Effects of 100 intermittent drop jumps protocol and high-intensity cycling exercise on MVCP

**Figure 2:**
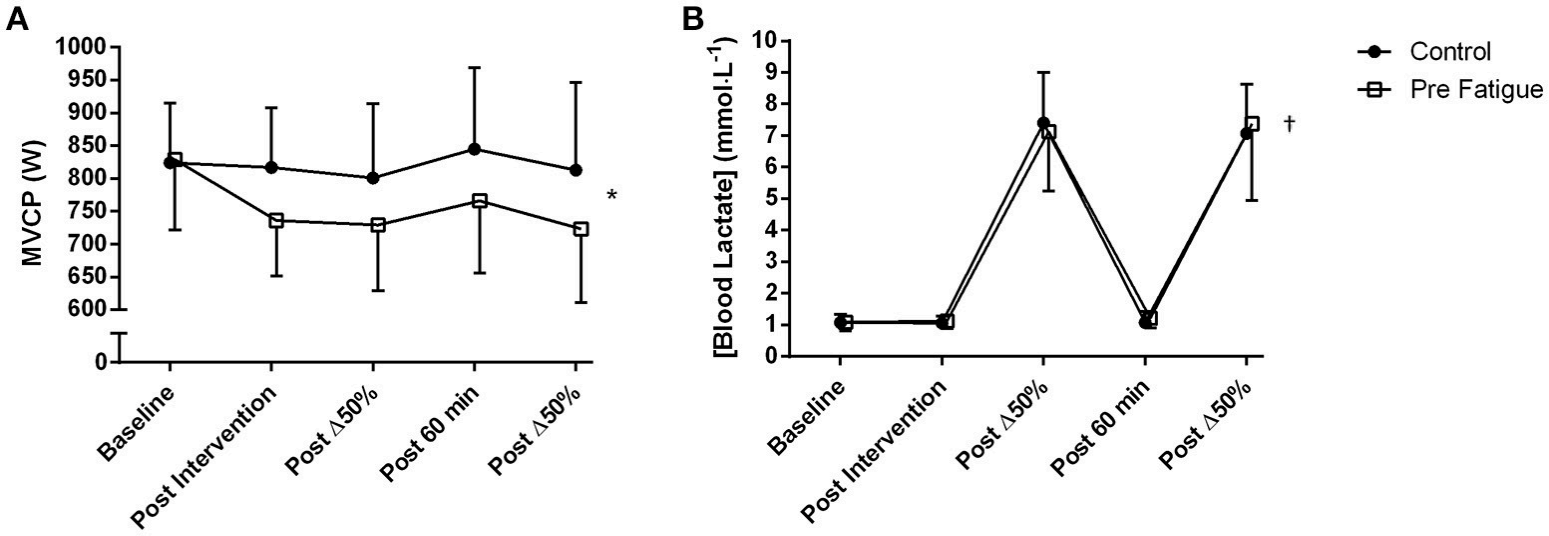
**Time course of change in (A) MVCP during 6 s “all-out” cycling tests; (B) blood lactate, from pre-fatigue and control conditions**. Fatigue condition consisted of 100 drop jumps over 33 min. Control condition consisted of 33 min rest. ^*^Significant condition × time point interaction (*P* < 0.05). ^†^Significant main effect of time (*P* < 0.05). Data are presented as means ± SD.

There was a significant condition × time interaction on MVCP (*P* = 0.03; Figure 2A). Tests of simple main effects of condition at each time point revealed no significant difference in MVCP at baseline. However, after experimental manipulation, MVCP was significantly lower in the pre-fatigue condition compared to the control condition at all time points (*P* = 0.02). Tests of simple main effects of time within each condition revealed that 6 min of cycling exercise at Δ50% did not induce a significant reduction in MVCP in either the pre-fatigue (*P* = 0.73) or the control *(P* = 0.54) condition.

### Effects of locomotor muscle fatigue on $\dot{\text{V}}{\text{O}}_{2}$ kinetics during high-intensity cycling exercise

**Figure 3:**
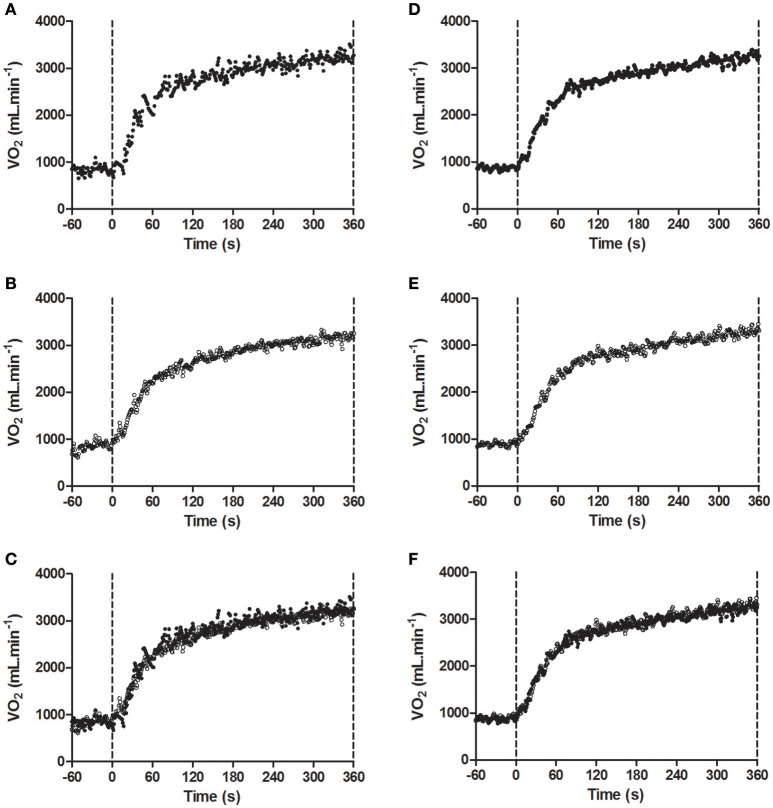
**Model fit of the $\dot{\text{V}}{\text{O}}_{2}$ kinetic response to exercise at Δ50% in (A) the pre fatigued experimental condition; (B) the control condition; (C) both experimental conditions overlaid for a representative participant, and (D) Bout 1; (E) Bout 2; (F) both bouts overlaid of the pre-fatigue condition for a representative participant**. Fatigue condition consisted of 100 drop jumps over 33 min. Control condition consisted of 33 min rest.

As shown in Table 1, $\dot{\text{V}}{\text{O}}_{2\text{b}}$ was not significantly different between conditions (*P* = 0.78). Figures 3A–C demonstrates a typical individual response in the control and pre fatigued conditions during high-intensity cycling exercise. Locomotor muscle fatigue had no effect on either the fundamental phase responses or the slow component during high-intensity cycling exercise. Specifically, neither the kinetic response (τ_p_, *P* = 0.72) nor the amplitude (A_p_, *P* = 0.75) of the fundamental phase were significantly different between conditions. The $\dot{\text{V}}{\text{O}}_{2^{\text{sc}}}$ and the trajectory (rate of the development of the $\dot{\text{V}}{\text{O}}_{2^{\text{sc}}}$) were also unaffected by locomotor muscle fatigue (*P* = 0.50 and *P* = 0.55, respectively). Figures 3D–F shows that the pattern of the $\dot{\text{V}}{\text{O}}_{2}$ kinetic response during high-intensity cycling exercise was similar between bouts 1 and 2 in the pre-fatigue condition.

**Table 1:** **Kinetic parameters of $\dot{\text{V}}{\text{O}}_{2}$ during exercise equivalent to Δ50% (mean power output of 242 ± 22 W)**.

	**Control**	**Pre-fatigue**
$\dot{\text{V}}{\text{O}}_{2\text{b}}$ (mL·min^−1^)	877 ± 58	867 ± 79
A_p_ (mL·min^−1^)	1885 ± 415	1945 ± 317
τ_p_ (s)	32 ± 9	30 ± 11
TD_p_ (s)	14 ± 4	12 ± 5
$\dot{\text{V}}{\text{O}}_{2^{\text{sc}}}$ (mL·min^−1^)	556 ± 223	464 ± 302
TD_sc_ (s)	106 ± 36	136 ± 66
Trajectory (mL·min^−2^)	131 ± 53	115 ± 49

*$\dot{\text{V}}{\text{O}}_{\text{2b}}$ represents the mean $\dot{\text{V}}{\text{O}}_{\text{2}}$ in the baseline period; A_p_, TD_p_, and τ_p_ represent the amplitude, time delay, and time constant, respectively, describing the fundamental or phase II increase in $\dot{\text{V}}{\text{O}}_{\text{2}}$ above baseline; and $\dot{\text{V}}{\text{O}}_{2^{\text{sc}}}$ and τ_sc_ represent the amplitude, and time constant describing the development of, the $\dot{\text{V}}{\text{O}}_{\text{2}}$ slow component, respectively. Values are mean ± SD*.

### Effects of locomotor muscle fatigue on blood lactate concentration, heart rate and RPE during high-intensity cycling exercise

There was no significant condition x time interaction (*P* = 0.48) and no significant main effect of condition *(P* = 0.75) on blood lactate concentration. There was, however, a significant main effect of time (*P* ≤ 0.01) with blood lactate concentration significantly increasing after each bout of high-intensity cycling exercise (*P* ≤ 0.01; Figure 2B).

**Figure 4:**
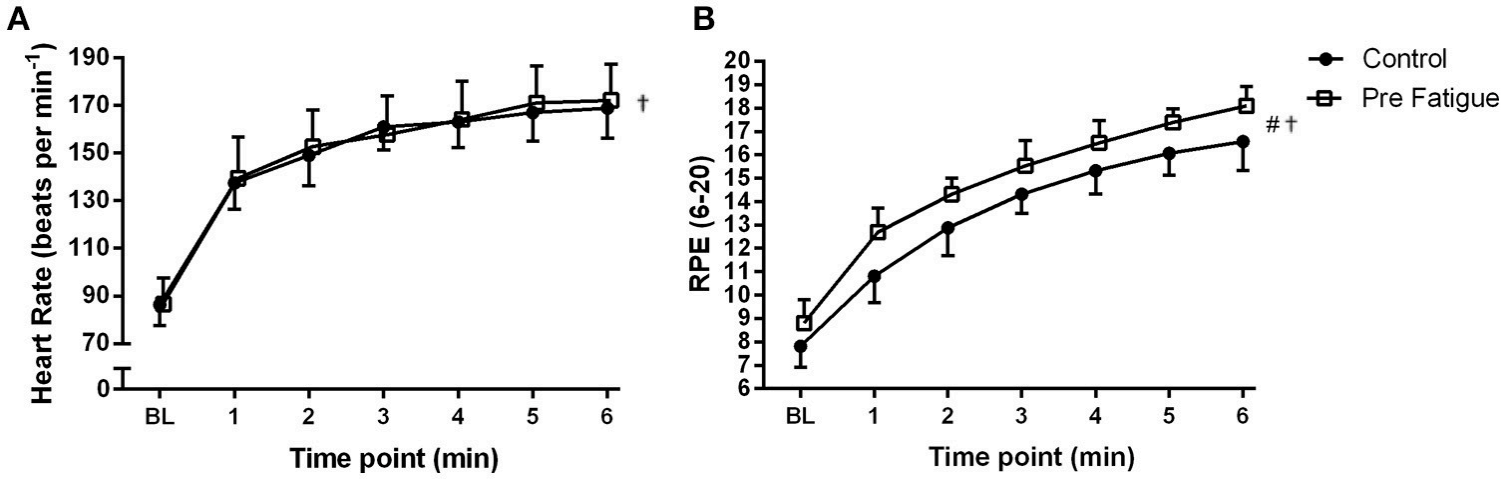
**Effects of experimental conditions on: (A) heart rate and (B) perceived exertion during high-intensity cycling at Δ50%**. Fatigue condition consisted of 100 drop jumps over 33 min. Control condition consisted of 33 min rest. BL = baseline measurement taken during 20 W cycling. ^†^Significant main effect of time (*P* < 0.05). ^#^Significant main effect of condition (*P* < 0.05). Data are presented as means ± SD.

There was no significant no significant condition x bout x time interaction (*P* = 0.89), condition x time interaction (*p* = 0.09), or bout x time interaction (*P* = 0.44). There was also no significant main effect of condition (*P* = 0.54), or exercise bout (*P* = 0.29) on heart rate. There was, however, a significant main effect of time (*P* ≤ 0.01) with heart rate progressively increasing during high-intensity cycling exercise. Heart rate responses are therefore presented as the grand average of both bouts in Figure 4A.

There was no significant no significant condition × bout × time interaction (*P* = 0.17) on RPE. The significant main effect of bout (*P* = 0.03) as well as significant condition x bout (*P* = 0.03) and bout × time (*P* = 0.02) interactions indicate that bout 2 was perceived as requiring more effort than bout 1. For sake of simplicity, RPE data are presented as the grand average of both bouts in Figure 4B. RPE increased over time in both conditions (condition × time interaction, *P* = 0.13; main effect of time, *P* ≤ 0.01) but was significantly higher in the pre-fatigue condition compared to the control condition (main effect of condition, *P* ≤ 0.01).

## Discussion

### Manipulation checks

As in previous studies (Skurvydas et al., 2002; Nielsen et al., 2005; Marcora et al., 2008) the 100 intermittent drop jumps protocol induced a significant reduction in locomotor muscle function with no significant increase in blood lactate concentration (Figure 2). The significant and prolonged reduction in MVCP (Figure 2A) would have necessitated additional muscle recruitment to meet the demands of the subsequent high-intensity cycling exercise bouts. The higher RPE measured in the pre-fatigue condition (Figure 4B) supports indirectly the assumption that a greater level of central motor command and muscle recruitment was required to produce the same power output as in the control condition (de Morree et al., 2012). Furthermore, blood lactate accumulation during the two bouts of high-intensity cycling exercise (a marker of metabolic stress in locomotor muscles) was not different between conditions (Figure 2B). Therefore, with reference to the work of Skurvydas et al. (2000), our data suggests that we were able to experimentally isolate the effects of additional recruitment to compensate for locomotor muscle fatigue from the effects of mechanisms like muscle metabolite accumulation, limited O_2_ or substrate availability, increased glycolysis and pH disturbance on $\dot{\text{V}}{\text{O}}_{2}$ kinetics during high-intensity cycling exercise.

### Locomotor muscle fatigue does not affect $\dot{\text{V}}{\text{O}}_{2}$ kinetics during high-intensity cycling exercise

There is a considerable body of literature suggesting an association between the $\dot{\text{V}}{\text{O}}_{2^{\text{sc}}}$ and the progressive recruitment of type II muscle fibers during aerobic exercise above LT (Saunders et al., 2000; Burnley et al., 2002; Krustrup et al., 2004a,b; Bernasconi et al., 2006; Endo et al., 2007). However, the link between the $\dot{\text{V}}{\text{O}}_{2^{\text{sc}}}$ and the progressive recruitment of Type II muscle fibers is not supported unanimously (Lucia et al., 2000; Scheuermann et al., 2001; Tordi et al., 2003; Zoladz et al., 2008). One of the difficulties is the poor validity of surface EMG to infer the level of muscle activation and the type of motor unit recruited, especially during dynamic contractions (Farina et al., 2004). Therefore, a more direct testing of the relationship between locomotor muscle fatigue and the $\dot{\text{V}}{\text{O}}_{2^{\text{sc}}}$ during aerobic exercise was required. The first to do so were Cannon et al. (2011) who found a significant correlation between the magnitude of locomotor muscle fatigue (measured by a reduction in MVCP) and the magnitude of the $\dot{\text{V}}{\text{O}}_{2^{\text{sc}}}$ measured during cycling at different exercise intensities. This finding lends some support to the “muscle fatigue and recruitment” hypothesis for the $\dot{\text{V}}{\text{O}}_{2^{\text{sc}}}$ observed during high-intensity aerobic exercise. However, in the same study, Cannon et al. (2011) found that locomotor muscle fatigue develops during the first 3 min of cycling exercise at Δ20 and Δ60%, with no further fatigue developing over the remaining 5 min of high-intensity cycling exercise even though a $\dot{\text{V}}{\text{O}}_{2^{\text{sc}}}$ is still evident. These data demonstrated for the first time dissociation between locomotor muscle fatigue and the development of the $\dot{\text{V}}{\text{O}}_{2^{\text{sc}}}$ during high-intensity cycling exercise. Therefore, the significant correlation between locomotor muscle fatigue and the $\dot{\text{V}}{\text{O}}_{2^{\text{sc}}}$ reported by Cannon et al. (2011) is most likely a spurious one, with both variables related to exercise intensity.

The $\dot{\text{V}}{\text{O}}_{2}$ kinetics data from the current experimental study provide experimental evidence that locomotor muscle fatigue (defined as an exercise-induced reduction in maximal voluntary force or power produced with the locomotor muscles) is not causally associated with the development of the $\dot{\text{V}}{\text{O}}_{2^{\text{sc}}}$ during high-intensity aerobic exercise. Indeed, none of parameters of the $\dot{\text{V}}{\text{O}}_{2}$ kinetics were affected by locomotor muscle fatigue (see Table 1, Figure 3). Specifically, the magnitude of $\dot{\text{V}}{\text{O}}_{2}$_sc_ during high-intensity cycling exercise was normal and not significantly different between the pre-fatigue and the control condition. We also found that, despite causing a significant $\dot{\text{V}}{\text{O}}_{2^{\text{sc}}}$, cycling for 6 min at Δ50% did not induce any significant reduction in MVCP in either the pre-fatigue or control condition. Clearly, as noted by Cannon et al. (2011), locomotor muscle fatigue might not necessarily be required for the development of the $\dot{\text{V}}{\text{O}}_{2^{\text{sc}}}$ during high-intensity aerobic exercise.

### Additional explanations for the $\dot{\text{V}}{\text{O}}_{2^{\text{Sc}}}$ during high-intensity aerobic exercise

As the $\dot{\text{V}}{\text{O}}_{2^{\text{sc}}}$ of the metabolic process have been suggested to arise from within the locomotor muscles (Jones et al., 2011), it is interesting to consider that pre-fatiguing the same muscles had no effect on altering the $\dot{\text{V}}{\text{O}}_{2}$ kinetic response to exercise above the LT; i.e., a reduced efficiency (or an increased O_2_ cost) of muscle contractions still occurred even though there were different levels of muscle fatigue between conditions at the start of exercise. The degree of blood lactate accumulation observed in our study clearly demonstrates that cycling exercise for 6 min at Δ50% was metabolically stressful for our participants. Thus, instead of greater recruitment of less efficient Type II fibers because of locomotor muscle fatigue, the explanation for the $\dot{\text{V}}{\text{O}}_{2^{\text{sc}}}$ observed during exercise above the LT may be challenges to cellular homeostasis sufficient to impair the efficiency of the working muscle fibers. Indeed, using a 3 min all-out high intensity cycling test, Vanhatalo et al. (2011) demonstrated that progressive fiber recruitment was not requisite for the development of the $\dot{\text{V}}{\text{O}}_{2^{\text{sc}}}$. Instead their results suggest that the $\dot{\text{V}}{\text{O}}_{2^{\text{sc}}}$ was likely generated by a higher muscle fiber oxygen cost per unit of external work done. Moreover, Ribeiro et al. (1986) found participants decreased mechanical power output in order to maintain a constant pulmonary $\dot{\text{V}}{\text{O}}_{2}$ during 40 min bouts at fixed percentages of $\dot{\text{V}}{\text{O}}_{2^{\text{max}}}$ (55–75%). Interestingly, the magnitude of the reduction in power output was linearly related to the relative metabolic power. However, in the current study, our data suggests that a reduction in efficiency and a $\dot{\text{V}}{\text{O}}_{2^{\text{sc}}}$ is still evident within pre fatigued muscles. Therefore, we argue that progressive muscle fatigue *per se*, may not be associated with muscle inefficiency and the development of the $\dot{\text{V}}{\text{O}}_{2^{\text{sc}}}$.

One possible mechanism for the $\dot{\text{V}}{\text{O}}_{2^{\text{sc}}}$ could be a reduction in efficiency of energy transfer within the mitochondria, specifically in the ratio between ADP phosphorylation and oxygen consumption (P/O ratio). Cannon et al. (2014) have shown that during high intensity exercise eliciting the $\dot{\text{V}}{\text{O}}_{2^{\text{sc}}}$, the tight coupling between ATP production $\dot{\text{V}}{\text{O}}_{2}$ seen during moderate intensity exercise is no longer evident. Indeed, changes in pH and high rates of glycolytic flux resulting from high intensity exercise have been suggested to reduce the P/O ratio (Özyener et al., 2001), suggesting that muscle inefficiency may result from both impaired ATP production and turnover. Another potential mechanism leading to increased mitochondrial uncoupling, reduced P/O ratio, and reduced efficiency is back leak of protons across the inner membrane without driving ATP-synthase (Rolfe and Brand, 1996). Alternatively, the $\dot{\text{V}}{\text{O}}_{2^{\text{sc}}}$ could also be caused by some mitochondrial ATP generation being used to reduce ROS generation within the cell (Brand, 2000). A high proton motive force that drives efficient ATP synthesis is associated with an additional ROS production. Proton leak across the mitochondrial membrane without driving ATP production may therefore assist in limiting the oxidative damage associated with high levels of ROS generated during the high intensity or prolonged aerobic exercise (Sahlin et al., 2010). High intensity aerobic exercise could also result in a reduction Gibbs free energy and necessitate an increased rate of ATP production to maintain steady-state SERCA ATPase function, and sarcoplasmic reticulum calcium flux. In turn, this could increase rate of ATP production and would thus reduce the efficiency of the muscular work during exercise (Grassi et al., 2015). Thus, there are potential mechanisms other than additional muscle recruitment to compensate for locomotor muscle fatigue which could explain the presence of the $\dot{\text{V}}{\text{O}}_{2^{\text{sc}}}$. However, the evidence for the possible mechanisms outlined above is sparse, and readers are referred to the review by Grassi et al. (2015) for a more in-depth discussion on this topic.

## Limitations

Due to the small sample size, the results of this study should be interpreted with caution. However, the sample size used in the current study replicates that of previously published works documenting the effects of muscle fatigue on oxygen uptake kinetics, such as Krustrup et al. (2004b) and Cannon et al. (2011). It could also be suggested that, given the exercise intensity of Δ50%, some of the participants might have achieved their $\dot{\text{V}}{\text{O}}_{2^{\text{peak}}}$ near the end of the 6 min cycling exercise bout in the control condition. If this was the case, then any inflation in the $\dot{\text{V}}{\text{O}}_{2^{\text{sc}}}$ caused by the pre-fatiguing exercise protocol might have been limited by the participants reaching their $\dot{\text{V}}{\text{O}}_{2^{\text{peak}}}$ prior to the end of the 6 min high-intensity cycling exercise bout. However, subjects reached ~94% of their $\dot{\text{V}}{\text{O}}_{2^{\text{peak}}}$ in both conditions, and so this was not a reason for the unchanged $\dot{\text{V}}{\text{O}}_{2}$ kinetic response.

It could also be argued that the MVCP test we used to quantify locomotor muscle fatigue requires the recruitment of all available muscle fibers, whereas 6-min cycling exercise bouts corresponding to Δ50% requiring about 3–4-fold less power do not necessitate the recruitment of the fastest type IIX muscle fibers. Therefore, the lack of effect of locomotor muscle fatigue on the $\dot{\text{V}}{\text{O}}_{2^{\text{sc}}}$ may be due to the fact that the pre-fatiguing exercise protocol induced fatigue in muscle fibers not needed to generate power during 6 min of high-intensity cycling exercise. However, there is strong evidence from analysis of muscle biopsies that type IIX muscle fibers are recruited within 1 min of cycling exercise performed at an exercise intensity even lower than the one employed in our study (Altenburg et al., 2007). Further muscle biopsy data from (MacAluso et al., 2012) demonstrate that jumping activities similar to those used in the current study affect muscle fibers from all subgroups, including type I and type IIa muscle fibers. Therefore, we are confident that our pre-fatiguing exercise protocol affected muscle fiber populations recruited during the following bouts of high-intensity cycling exercise. Moreover, it could be suggested that the 100 intermittent drops jumps may have induced a muscle PCr overshoot which could affected the $\dot{\text{V}}{\text{O}}_{2}$ on-kinetics. However, Nielsen et al. (2005) demonstrate that PCr concentration was unchanged in a muscle biopsy taken 13–15 min following the same intermittent jumping protocol as used in this study.

The fact that we performed two replications of the exercise transition for the determination of $\dot{\text{V}}{\text{O}}_{2}$ kinetic response with the 2nd bout not being preceded by the pre-fatiguing exercise protocol may also be considered a limitation. However, due to the long lasting effects of eccentric exercise on muscle function (Twist and Eston, 2005), we deemed unnecessary to repeat the pre-fatiguing exercise protocol before the 2nd bout of high-intensity cycling exercise. The validity of this choice is corroborated by the prolonged reduction in MVCP induced by our pre-fatiguing exercise protocol (Figure 2A). Furthermore, data presented in Figure 3B demonstrates that that the pattern of the $\dot{\text{V}}{\text{O}}_{2}$ kinetic response during high-intensity cycling exercise was similar between bouts 1 and 2 in the pre-fatigue condition.

## Conclusions

By pre-fatiguing the locomotor muscles without inducing metabolic stress, this study has demonstrated experimentally that locomotor muscle fatigue can be dissociated from the $\dot{\text{V}}{\text{O}}_{2^{\text{sc}}}$ observed during aerobic exercise above LT. Therefore, additional muscle recruitment to compensate for locomotor muscle fatigue might not be the main driver of the $\dot{\text{V}}{\text{O}}_{2^{\text{sc}}}$ during high-intensity aerobic exercise. Further research is required to test the hypothesis that an increased ATP and/or O_2_ cost of power production in the active muscle fibers is responsible for the development of the $\dot{\text{V}}{\text{O}}_{2^{\text{sc}}}$ during exercise above LT.

## Author contributions

JH, AA, and SM developed the study methodology, AA, GC, RC collected the data, all authors contributed to analysis of the results. JH and SM drafted the manuscript, and all authors reviewed and revised the work. All authors reviewed the final manuscript and approved it for submission.

### Conflict of interest statement

The authors declare that the research was conducted in the absence of any commercial or financial relationships that could be construed as a potential conflict of interest.
